# Repair of UVB-induced DNA damage is reduced in melanoma due to low XPC and global genome repair

**DOI:** 10.18632/oncotarget.10902

**Published:** 2016-07-28

**Authors:** Timothy Budden, Ryan J. Davey, Ricardo E. Vilain, Katie A. Ashton, Stephen G. Braye, Natalie J. Beveridge, Nikola A. Bowden

**Affiliations:** ^1^ Hunter Medical Research Institute and Faculty of Health, University of Newcastle, Callaghan, NSW, Australia; ^2^ Melanoma Institute of Australia, Camperdown, NSW, Australia; ^3^ Sydney Medical School, University of Sydney, Sydney, NSW, Australia; ^4^ Hunter Area Pathology Service, Pathology North, John Hunter Hospital, Newcastle, NSW, Australia

**Keywords:** melanoma, UVB, global genome repair, XPC, nucleotide excision repair

## Abstract

UVB exposure leads to DNA damage, which when unrepaired induces C>T transitions. These mutations are found throughout the melanoma genome, particularly in non-transcribed regions. The global genome repair (GGR) branch of nucleotide excision repair (NER) is responsible for repairing UV-induced DNA damage across non-transcribed and silent regions of the genome. This study aimed to examine the relationship between UVB and GGR in melanoma. DNA repair capacity and relative expression of NER in melanocytes and melanoma cell lines before and after treatment with UVB was quantified. Transcript expression from 196 melanomas was compared to clinical parameters including solar elastosis and whole transcriptome data collected. Melanoma cell lines showed significantly reduced DNA repair when compared to melanocytes, most significantly in the S phase of the cell cycle. Expression of GGR components XPC, DDB1 and DDB2 was significantly lower in melanoma after UVB. In the melanoma tumours, XPC expression correlated with age of diagnosis and low XPC conferred significantly poorer survival. The same trend was seen in the TCGA melanoma dataset. Reduced GGR in melanoma may contribute to the UV mutation spectrum of the melanoma genome and adds further to the growing evidence of the link between UV, NER and melanoma.

## INTRODUCTION

Exposure to ultraviolet radiation (UV) is a major risk factor for melanoma development [1]. The main effect of UVB is DNA damage in the form of two individual DNA photoproducts, cyclobutane pyrimidine dimers (CPDs) and 6-4 photoproducts (6-4 PPs). The photoproducts create a bulky lesion that distorts the DNA helix and can halt transcription and DNA replication [2, 3].

Unrepaired photoproducts can lead to mutations, most frequently C > T or CC > TT transitions at dipyrimidine sites, commonly referred to as UV-fingerprint mutations [4, 5]. In recent genome and exome sequencing of melanoma cell lines and tumours samples [6–8], the vast majority of mutations found were UV-fingerprint mutations. The mutations occur more frequently in untranscribed regions of the genome [8] and have recently been found overrepresented in active promoters of the melanoma genome [9, 10].

This mutation spectrum is indicative of unrepaired UV-induced DNA damage [4]. Belanger and colleagues [11] recently reported S-phase deficiency in repair of 6-4 PPs and CPDs in melanoma cell lines, which was attributed to depletion of ATR, a DNA repair protein that acts downstream of DNA damage recognition by nucleotide excision repair (NER) [12, 13]. The NER pathway is responsible for removing UV photoproducts [14]. There are two branches of damage recognition in NER, global genome repair (GGR) and transcription coupled repair (TCR). TCR functions with high priority to repair damage that is in actively transcribed genes [15]. GGR is not dependent on transcription and repairs lesions across the entire genome including both strands of active and silent genes, and non-transcribed regions. GGR involves DNA damage recognition complexes XPC and UV-DDB (composed of DDB1 and DDB2) binding directly to the DNA distortion caused by the UV lesion, then signal for repair. We have previously quantified NER transcripts in melanocytes and melanoma cell lines after treatment with the DNA-helix distorting agent, cisplatin. Expression of GGR components, *XPC, DDB1* and *DDB2* significantly increased in response to cisplatin in melanocytes but this increase was noticeably absent in melanoma [16]. In addition, [9] used a large-scale genome informatics approach to identify differential NER is responsible for a high UVR mutation load in the promoters of the melanoma genome.

Taken together, the epidemiological and genomic features of melanoma indicate that high UVR and dysfunctional NER, particularly the GGR component, may play complimentary roles in the development of melanoma. In this study, we quantified DNA repair capacity and NER in melanoma and melanocytes after UVB. To further investigate the S-phase deficiency previously reported [11] we quantified cell cycle phase specific repair and investigated a subset of NER in melanocyte and melanoma cell lines before and after UVB. The transcript levels of *XPC, DDB1* and *DDB2* were also quantified in melanoma tumours and compared to clinical information. Whole transcriptome analysis was conducted to further investigate the biological features of melanomas with high and low *XPC.*

## RESULTS

**Figure 1:**
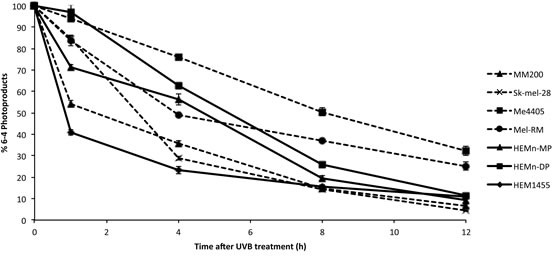
Repair of 6-4 photoproducts in melanoma and melanocyte cell lines after 650J/m^2^ UVB: Geometric mean fluorescence (FITC) was normalized to baseline to calculate the percentage of 6-4 PPs remaining at each timepoint Points are mean of triplicates of three individual experiments, bars = SE.

To determine whether NER is reduced in melanoma we first quantified the removal of the UV photoproducts 6-4 PPs and CPDs, in melanoma and melanocyte cell lines after UVB treatment. 6-4 PPs were induced by 650J/m^2^ UVB in all cell lines (Figure 1). At 12 hours almost 90% of 6-4 PPs were repaired in intact melanocytes (Figure 1), while melanoma cell lines Me4405 and Mel-RM retained over 30% of 6-4 PPs (Figure 1). MM200 and Sk-mel-28 obtained similar levels of repair to melanocytes by 12 hours. Repair was significantly lower at 12 hours (*p* < 0.0005) for Me4405 and Mel-RM.

**Figure 2:**
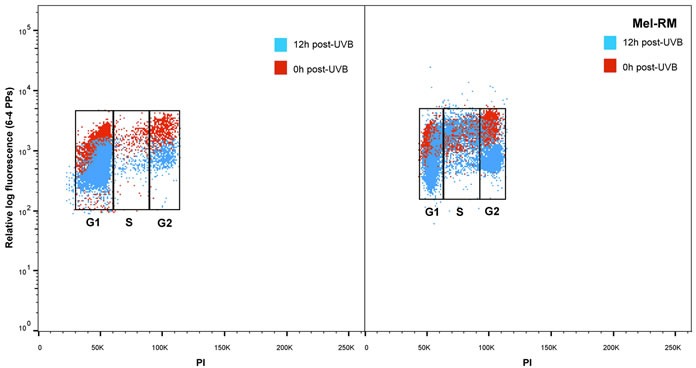
Repair of 6-4 photoproducts in melanoma and melanocytes in individual phases of the cell cycle: Bivariate distributions of 6-4 PPs *versus* DNA content (PI) in **a.** melanocytes (HEMn-MP) and **b.** melanoma cell line (Mel-RM) immediately (red dots) and 12 hours post-UVB (blue dots). **c.** All cell lines were divided into individual cell cycle phases, and repair of 6-4 PPs was measured in each individual phase. Points are mean of triplicates of three individual experiments, bars = SE.

Analysis by cell cycle stage, confirmed that overall in melanocytes 6-4PPs were repaired in every phase of the cell cycle (Figure 2a and 2c). For all the melanoma cell lines a similar pattern of delayed repair was observed for the G1 and G2 phases. But in the S phase of melanoma cell lines 6-4PP repair was significantly reduced at 8 and 12 hours (Figure 2b and 2c). In contrast to average 6-4PP removal (Figure 1) where only Me4405 and Mel-RM displayed reduced repair, S phase repair was reduced in all of the melanoma cell lines, and did not reach repair levels of melanocytes (Figure 2c). To ensure the repair deficiency was not due to high cell cycle rate in the melanoma cell lines BrdU was used to observe movement through the cell cycle phases across the timeseries. There was no increase in BrdU+ cells in G1, S or G2 phase at 12 or 24 hours (Supplementary Figure S1), indicating that cells did not progress through the cell cycle during the 12 hours.

**Figure 3:**
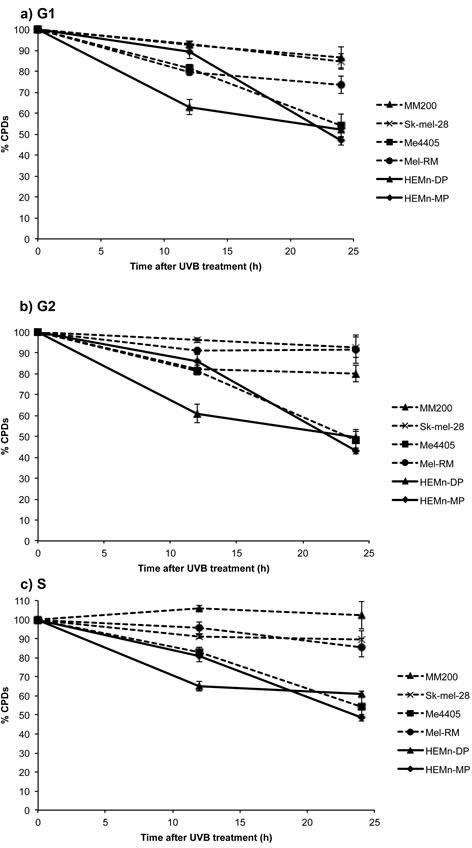
Repair of CPDs in melanoma and melanocytes in individual phases of the cell cycle: All cell lines were divided into individual cell cycle phases, using propidium iodide staining and the repair of CPDs was measured in each individual phase Significance was calculated between levels of CPDs in melanocytes and melanoma at each time point. Points are mean of triplicates of three individual experiments, bars = SE.

Repair of CPDs was also reduced in melanoma compared to melanocytes, but at a much slower rate than 6-4PP repair. Significantly higher levels of CPDs were still present in all melanoma cell lines, except Me4405, 24 hours post-UVB (Figure 3). The repair deficiency was present in all phases of the cell cycle, with the S-phase specific deficiency commencing at 12 hours (Figure 3c). Interestingly, the p53 null melanoma cell line, Me4405 displayed similar CPD repair to melanocytes in all phases of the cell cycle, despite being deficient in 6-4PP repair. Altogether, these results indicate repair of both 6-4PPs and CPDs is significantly impaired in melanoma cell lines, particularly in S-phase, compared to melanocytes.

**Figure 4:**
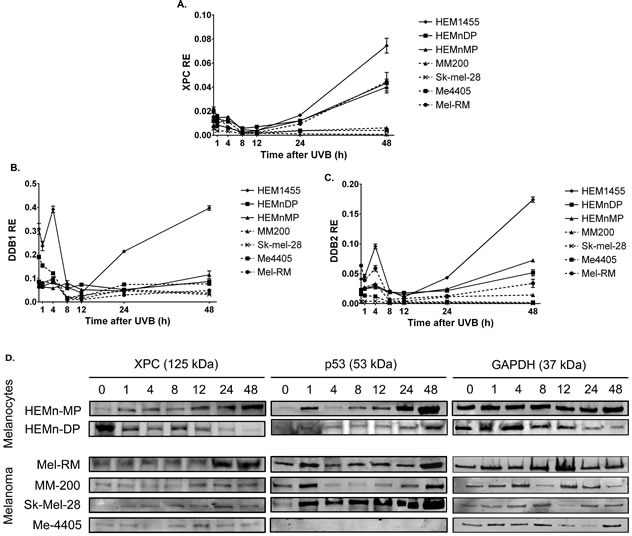
Expression of GGR and p53 in melanocytes and melanoma cell lines after treatment with 650J/m UVB **a.**-**c.** relative expression (RE) GGR transcripts *XPC*, *DDB1*, and *DDB2* in melanocytes and melanoma cell lines. Points are mean of triplicates of three individual experiments, bars = SE, **d.** Western blot of XPC and p53 for all cell lines before and after UVB. Blots are representative of duplicate blots run for all proteins and cell lines.

To investigate the cause of the reduced 6-4PP and CPD removal in melanoma, the expression of all NER transcripts was quantified in melanocyte and melanoma cell lines after 650J/m^2^ UVB. Expression of the DNA damage recognition GGR components *XPC, DDB1* and *DDB2* was significantly higher in melanocytes than melanoma from 4 or 8 to 48 hours after UVB irradiation (Figure 4a-4c). In contrast all three transcripts were not significantly induced in melanoma cell lines, except *XPC* in Mel-RM at 48 hours, after UVB treatment. The absence of functional p53 in Me4405 and Sk-mel-28 did not result in a significant difference in *XPC, DDB1* or *DDB2* transcript levels when compared to MM200 and Mel-RM with wildtype p53, all 4 melanoma cell lines displayed similar reduced transcript levels. The attenuation of post-UVB XPC expression was confirmed at the protein level in all melanoma cell lines, except Mel-RM (Figure 4d and Table 1). The melanocyte cell line HEMn-DP did not induce XPC protein, but instead displayed a high baseline XPC level that was relatively stable for the 48 hours post-UVB (Table 1).

**Table 1:** XPC and p53 protein expression in melanocytes and melanoma after UVB

		Time (hours post UVB-irradiation)						
		0	1	4	8	12	24	48
XPC	HEMn-MP	1±0.0.15	1±0.0.2	2.09±0.19	1.87±0.12	2.02±0.29	3.60±0.36	3.97±0.12
	HEMn-DP	1±0.0.19	0.88±0.0.21	0.82.±0.11	1.08±0.17	1.01±0.20	0.82±0.19	0.7±0.19
	Mel-RM	1±0.0.15	1.32±0.12	1.40±0.13	1.10±0.12	1.04±0.14	2.80±0.28	2.42±0.18*
	MM-200	1±0.0.11	1.2±00.11	1.60±0.14	1.50±0.11	0.90±0.21	1.80±0.23	2.20±0.23*
	Sk-Mel-28	1±0.0.15	1.3±00.12	1.20±0.11	1.80±0.13	1.90±0.25	2.04±0.21	1.48±0.13^#^
	Me-4405	1±0.0.14	1.22±0.1	1.26±0.08	1.39±0.12	1.26±0.16	1.98±0.13	1.82±0.1^#^
p53	HEMn-MP	1±0.0.13	2.2±0.27	1.20±0.18	1.04±0.19	1.8±0.37	2.70±0.33	3.10±0.68
	HEMn-DP	1±0.0.15	0.71±0.0.22	0.72±0.123	0.7±0.11	0.81±0.24	1.89±0.21	4.23±0.13
	Mel-RM	1±0.0.13	1.56±0.32	1.02±0.1	0.96±0.07	2.02±0.26	2.52±0.25	3.09±0.15*
	MM-200	1±0.0.23	1.36±0.12	1.21±0.11	0.65±0.12	0.61±0.07	0.41±0.04	2.86±0.34*
	Sk-Mel-28	1±0.0.15	2.20±0.19	1.20±0.16	1.05±0.07	1.80±0.23	2.70±0.27	3.10±0.41*
	Me-4405	0	0	0	0	0	0	0

* *p*<0.05,

# *p*<0.01

p53, a previously reported regulator of GGR, was induced in all cell lines post-UVB, with the exception of Me4405 (p53 null) (Figure 4d), thus indicating that p53 is not responsible for the reduced GGR. Interestingly, at baseline XPC and DDB2 were expressed at similar levels in melanocyte and melanoma cell lines. Components of the TCR damage recognition arm and the down-stream convergent NER pathway were not consistently higher or lower in melanoma compared to melanocytes at any timepoint post-UVB (Table 2).

**Table 2:** Expression of transcription coupled repair and nucleotide excision repair transcripts in melanocytes and melanoma after UVB

Time (h)	Cell type	ERCC6 (CSB)	ERCC8 (CSA)	XPA	RPA1	RPA2	ERCC1	ERCC2 (XPD)	ERCC3 (XPB)	ERCC4 (XPF)	ERCC5 (XPG)
0	Melanocytes	1.95	0.19	0.93	4.7	0.14	12.96	3.54	2.95	1.21	0.56
	Melanoma	0.62^‡^	0.31*	0.86	6.23	2.95^‡^	11.54	1.55^‡^	3.87	1.32	2.79^‡^
1	Melanocytes	1.38	0.11	0.48	4.7	2.01	11.3	1.91	2.3	0.96	1.2
	Melanoma	0.15^‡^	0.08	0.48	6.23	4.05^‡^	9.59	1.71	2.65	0.50*	0.69
4	Melanocytes	0.12	0.05	0.27	4.05	1.34	17.68	4.77	2.34	0.12	0.07
	Melanoma	0.17	0.11*	0.82^#^	6.3^#^	4.63^‡^	11.64^#^	1.87^‡^	3.13	0.5^#^	0.91^‡^
24	Melanocytes	0.15	0.04	0.12	3.32	0.92	9.19	1.42	0.73	0.04	0.04
	Melanoma	0.06*	0.04	0.25	2.67*	1.78^‡^	6.06^#^	0.73^#^	0.65	0.12	0.27^‡^
48	Melanocytes	0.76	0	0.45	4.91	1.98	22.96	2.82	2.49	0.27	0.41
	Melanoma	0.28#	0.04^‡^	0.41	2.47^‡^	1.16^#^	6.13^#^	0.98^#^	1.16*	0.28	0.84

* *P* < 0.05,

# *P* < 0.01,

‡ *P* < 0.001

Altogether this data confirms that melanoma cells have a significant lack of repair in S-phase due to limited induction of GGR. 6-4 PPs and CPDs are repaired exclusively by NER, therefore the lack of repair further confirms melanomas are deficient in NER, in particular GGR.

To further investigate the GGR deficiency in melanoma and its possible clinical implications we investigated *XPC, DDB1* and *DDB2* expression in a cohort of 196 primary and metastatic melanomas (clinical details in Supplementary Table 1). Transcripts were detectable in 157 of the 196 melanomas, the remaining 39 were excluded from analyses. To investigate the clinical relevance of GGR transcripts we undertook correlation analysis with the clinical parameters and Kaplin-Meier survival analysis for *DDB1, DDB2* and *XPC.*

**Figure 5:**
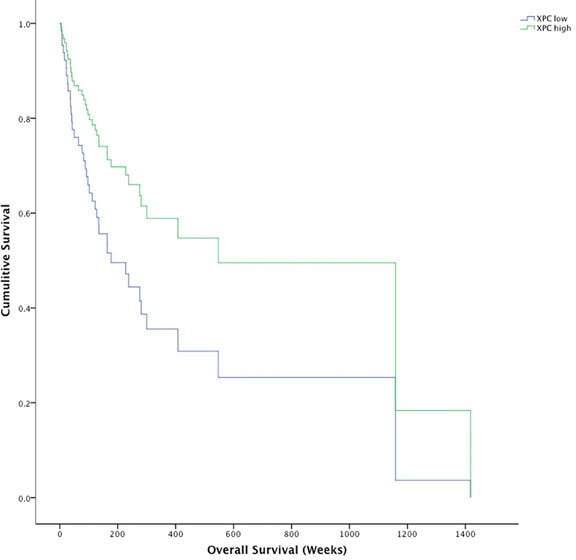
Kaplin-Meier survival plot for XPC high melanomas (green line) and XPC low melanomas (blue line) Survival for XPC high melanomas was significantly longer (733.5 weeks, 95% CI 456.8 - 1010.3) than XPC low melanomas (454.7 weeks, 95% CI 262.2 - 647.2) *p = 0.037.

Solar elastosis scoring (0 = none to 3 = severe) around the site of the primary melanoma significantly correlated with *DDB1* (r_s_ = 0.249, *p* = 0.043). *XPC* showed a trend towards correlation with solar elastosis but it did not reach significance and *DDB2* did not correlate. Further analysis of GGR transcripts in a larger cohort with more solar elastotic diversity will address this inconclusive finding. More interestingly, *XPC* correlated with age of diagnosis in our tumour cohort (r_s_ = 0.213, *p* = 0.037) indicating older age of diagnosis had higher *XPC* transcript levels. In addition, melanomas expressing lower than the mean XPC (XPC-) had 164.14 (95% CI 115.82 - 212.46) median weeks survival compared to 547.29 (95% CI 142.12 - 952.45) median weeks survival for those with high XPC expression (XPC+) (χ^2^ = 4.34 *p* = 0.037) (Figure 5). When corrected for primary vs metastatic disease and Breslow thickness the adverse impact on survival associated with XPC was still observed (HR = 2.972, *p* = 0.028). Survival analysis of melanomas with the lowest 10^th^ percentile and highest 10^th^ percentile XPC transcript expression in the TCGA melanoma dataset resulted in a similar trend towards poor survival in relation to low XPC transcript expression; TCGA 552.7 weeks for XPC+ compared to 221.1 weeks for XPC-, but it did not reach significance (*p* = 0.2). *DDB1* and *DDB2* were not significantly different across different stages or Breslow thickness.

Transcriptome analysis was used to investigate biological processes that differed between the XPC- and XPC+ melanomas. Mann-Whitney unpaired test was used to identify 1836 transcripts with significantly different expression between the XPC- and XPC+ groups. 973 transcripts were expressed higher in the XPC- group and 863 transcripts were expressed higher in the XPC+ group. In both the XPC- and XPC+ groups there was an over-representation of highly expressed transcripts involved in cell cycle, apoptosis and stress response. Perturbation of these biological processes is common in all cancer types [17, 18] and was consistent across all tumours in this study. Transcripts expressed significantly higher in the XPC- group are involved in DNA repair and DNA damage response, particularly double-strand break repair. Compensatory mechanisms for reduced NER may be occurring in this sub-group of melanomas. Protein kinase regulation and activity was also over-represented in this group (Table 3), further supporting the data indicating melanomas with higher kinase activity due to activating kinase mutations require UVR-induced mutations to accelerate melanomagenesis [19]. There was an over-representation of immune response and anatomical/structural processes in the XPC+ group (Table 4), but no kinase activity/regulation or DNA repair.

**Table 3:** Biological processes over-represented in XPC low melanomas

Biological Process	Gene Ontology Set Name	Description	# Genes in Gene Set (K)	# Genes in Overlap (k)	k/K	p-value	FDR q-value
Cell cycle	Mitotic cell cycle	GO:0000278	153	23	0.1503	4.37E-14	6.68E-12
	Cell cycle	GO:0007049	315	31	0.0984	2.67E-13	3.62E-11
	Mitosis	GO:0007067	82	16	0.1951	5.22E-12	5.80E-10
	M phase of mitotic cell cycle	GO:0000087	85	16	0.1882	9.32E-12	9.49E-10
	Cell cycle phases	GO:0022403	170	21	0.1235	2.63E-11	2.29E-09
	Cell cycle process	GO:0022402	193	22	0.114	4.40E-11	3.58E-09
	Regulation of cell cycle	GO:0051726	182	20	0.1099	6.42E-10	3.92E-08
	Regulation of mitosis	GO:0007088	41	9	0.2195	8.21E-08	3.34E-06
	Cell proliferation	GO:0008283	513	33	0.0643	3.74E-09	1.99E-07
Apoptosis	Regulation of apoptosis	GO:0042981	341	21	0.0616	4.92E-06	1.09E-04
	Apoptosis	GO:0006915	431	23	0.0534	1.83E-05	3.24E-04
Stress/damage response	Response to DNA damaging stimulus	GO:0006974	162	16	0.0988	1.46E-07	5.40E-06
	DNA repair	GO:0006281	125	14	0.112	1.84E-07	6.24E-06
	Response to stress	GO:0006950	508	29	0.0571	4.03E-07	1.26E-05
	Response to endogenous stimulus	GO:0009719	200	17	0.085	5.21E-07	1.55E-05
	Transcription from RNA polymerase II promoter	GO:0006366	457	28	0.0613	1.54E-07	5.52E-06
Kinase activity	Protein kinase activity	GO:0004672	285	17	0.0596	5.69E-05	8.47E-04
	Regulation of protein kinase activity	GO:0045859	155	12	0.0774	6.03E-05	8.76E-04
	Kinase activity	GO:0016301	369	23	0.0623	1.45E-06	3.75E-05

**Table 4:** Biological processes over-represented in XPC high melanomas

Biological Process	Gene Set Name	GO term	# Genes in Gene Set (K)	# Genes in Overlap (k)	k/K	*p*-value	FDR *q*-value
Cell proliferation	Cell proliferation	GO:0008283	513	34	0.0643	6.80E-11	5.19E-09
	Regulation of cellular proliferation	GO:0042127	308	20	0.0649	3.28E-07	1.00E-05
	Negative regulation of cell proliferation	GO:0008285	156	12	0.0769	1.37E-05	2.46E-04
Apoptosis	Apoptosis	GO:0006915	431	23	0.0534	1.38E-06	3.24E-05
	Regulation of apoptosis	GO:0042981	341	18	0.0528	2.18E-05	3.69E-04
Stress/damage response	Response to oxidative stress	GO:0006979	46	7	0.1522	1.08E-05	2.15E-04
	Response to stress	GO:0006950	508	23	0.0453	2.00E-05	3.44E-04
	Response to chemical stimulus	GO:0042221	314	17	0.0541	2.66E-05	4.27E-04
	Negative regulation of transcription DNA dependent	GO:0045892	130	11	0.0846	1.27E-05	2.31E-04
	Negative regulation of RNA metabolic process	GO:0051253	132	11	0.0833	1.47E-05	2.60E-04
Immune response	Immune system process	GO:0002376	332	26	0.0783	1.10E-10	7.89E-09
	Immune response	GO:0006955	235	21	0.0894	6.28E-10	3.65E-08
	Cytokine binding	GO:0019955	48	8	0.1667	1.23E-06	2.94E-05
	Adaptive immune response	GO:0002250	25	5	0.2	5.18E-05	7.53E-04
Anatomical/structural processes	Anatomical structure development	GO:0048856	1013	45	0.0444	4.50E-09	1.96E-07
	Cytoskeletal protein binding	GO:0008092	159	14	0.0881	5.33E-07	1.55E-05
	Organellle organisation and biogenesis	GO:0006996	473	25	0.0529	5.79E-07	1.61E-05
	Cytoskeleton orgnization and biogenesis	GO:0007010	208	14	0.0673	1.25E-05	2.31E-04
	Cellular component assembly	GO:0022607	298	16	0.0537	5.01E-05	7.45E-04
	Membrane organization and biogenesis	GO:0016044	135	10	0.0741	9.69E-05	1.31E-03
	Vesicle mediated transport	GO:0016192	194	12	0.0619	1.15E-04	1.51E-03
Protein function/modification	Phosphoprotein phosphatase activity	GO:0004721	81	11	0.1358	1.11E-07	3.76E-06
	Protein modification process	GO:0006464	631	31	0.0491	1.34E-07	4.30E-06
	Post translational protein modification	GO:0043687	476	25	0.0525	6.49E-07	1.76E-05
	Protein complex assembly	GO:0006461	167	13	0.0778	5.31E-06	1.12E-04
	Nucleoside triphosphatase activity	GO:0017111	212	13	0.0613	6.62E-05	9.29E-04
	Phosphorylation	GO:0016310	313	16	0.0511	8.92E-05	1.22E-03
	RAS protein signal transduction	GO:0007265	66	7	0.1061	1.17E-04	1.52E-03

## DISCUSSION

In this first study to investigate NER in melanoma after UVB we have confirmed that GGR is reduced in melanoma by showing delayed repair of both 6-4 PPs and CPDs, particularly in the crucial S-phase of the cell cycle, and lack of induction of *XPC, DDB1* and *DDB2* after UVB. Reduced *XPC* was also found associated with earlier onset of disease and poorer survival in a cohort of predominantly high sun exposed melanomas.

A previous investigation of repair of 6-4 PPs and CPDs found that approximately 80% of melanomas exhibited reduced repair, specifically in S-phase. However, GGR and NER transcripts and/or proteins were not investigated. A lack of phosphorylated histone H2AX was reported and suggested the defect in repair was due to decreased ATR signalling [11]. The decreased ATR signalling may be a result of the GGR deficiency we observed. ATR is activated in response to UV-induced DNA damage and initiates a phosphorylation cascade that can lead to cell cycle arrest and DNA repair [20]. XPC and DDB2 are required for activation of this pathway upstream of ATR. Processing of DNA photoproducts by XPC and DDB2 is required to form single stranded, unwound DNA that has been coated with RPA, which then recruits ATR [12, 13]. Indeed, human fibroblasts defective in XPC or DDB2 are unable to induce checkpoint activation following UV irradiation [21, 22]. Recent studies have also shown that XPC and DDB2 are an upstream requirement for ATR recruitment and activation at UV-induced DNA damage through physical interaction between these proteins [23]. Abrogation of ATR and ATM does not affect the recruitment of XPC and DDB2 to the damage site and as such did not affect NER efficiency. Altogether these studies confirm that GGR is tightly linked to the DNA damage response and checkpoint activation pathway, upstream of ATR. Previous literature and the reduced GGR transcript/proteins in our study, suggests that the decreased ATR signalling observed by Belanger and colleagues is due to a lack of lesion processing by reduced XPC and DDB2.

In addition to the well-characterised NER deficient skin cancer disorder xeroderma pigmentosum, the relationship between XPC deficiency and carcinogenesis after UV radiation has been described for melanoma and squamous cell carcinomas. An XPC−/−Ink4a-Arf−/− double knockout mouse model developed significantly more melanomas after a single neonatal dose of UVB than Wildtype or single knockout mice [24] and XPC expression was lost in up to 59% of SCCs via chromosome 3p loss or XPC mutation [25]. Interestingly, analysis of XPC−/− SCCs did not reveal a high UVR mutation load in the promoter regions of the genome which was in contrast to that seen in UV-induced melanoma genomes. It was concluded the promoter mutation load was due to differential NER rather than complete absence of NER [9], which was supported by the results of our study. Further to this, Sabarinathan, Mularoni [10] demonstrated using bioinformatics analysis that the rate of somatic mutations in melanomas is highly increased at active transcription factor binding sites and nucleosome embedded DNA, caused by a decrease of the levels of nucleotide excision repair (NER) activity. Once again, the functional assays and *in vitro* analysis of NER used in our study have supported these findings. Taken together, the recent melanoma genome bioinformatics studies [9, 10], transcriptome analysis and functional *in vitro* data presented herein confirm that NER deficiency is a key feature of melanoma.

In contrast to these findings, a study by Gaddameedhi et al. (2010) concluded that there was no difference in NER capacity between melanocytes and melanoma cell lines. Repair of CPDs was quantified up to 12 hours post-UVC by a slot-blot assay. The flow cytometry method used by Belanger et al. (2014) and in our study utilises a different quantitative measurement of residual CPDs than slot blot assays which may account for the differing conclusions of these studies. In our study, one melanocyte cell line, HEMn-DP, displayed a lack of XPC protein induction that reflected a reduced S phase repair of 6-4PPs when compared to the other melanocyte cell lines. Although HEMn-DP displayed the lowest levels of melanocyte S-phase repair, the levels were still higher than the melanoma cell lines. This may be due to the presence of high baseline XPC protein in HEMn-DP. Similarly, the repair of CPDs occurred more rapidly in the HEMn-DP cell line at 12hrs despite no induction of XPC protein. After 12 hours the CPD repair plateaued for this cell line, we postulate this is due to partial depletion of baseline XPC protein at 12 hours. Despite showing the lack of XPC protein induction across the 48hours post-UVB rendering HEMn-DP an outlier compared to the other melanocyte cell lines, the baseline expression of XPC protein was high enough in HEMn-DP to induce higher CPD and 6-4PP repair than in the melanoma cell lines. The variability in the protein levels seen in this study may be due to the low abundance of the nuclear XPC protein. A more accurate method of quantification such as multiple reaction monitoring (MRM) mass spectrometry could be used for future studies to accurately quantify the XPC protein levels after UVB.

Although there is evidence that p53 regulates the rate of CPD repair [26, 27], there are many studies that conclude p53 is not required for induction of XPC, DDB2 and subsequent repair of 6-4PPs [16, 26, 28, 29]. We observed almost comparable CPD removal in Me4405 p53 null melanoma cells and p53 proficient melanocytes, indicating that p53 may not regulate CPD removal in melanoma as wildtype p53 melanoma cell lines had much less CPD repair across 24 hours. PTEN has also been reported to regulate GGR, particularly XPC in keratinocytes after UVB radiation [30]. Although not the focus of the current study, PTEN may play a role in regulation of GGR and warrants further investigation in melanoma.

GGR proteins have functions outside of DNA repair that further their role in protecting against cancer. Both XPC and DDB2 (through the UV-DDB complex) are involved in apoptosis and cell cycle regulation in addition to the DNA damage response after UV irradiation. In brief, DDB2, through its role in the Cul4A ubiquitin complex regulates levels of p21 by triggering its ubiquitination and degradation [31, 32]. After UV exposure p21 accumulates, leading to cell cycle arrest. Degradation via DDB2 is required to allow for successful induction of apoptosis [33, 34]. XPC deficient cells exhibit an absence of caspase-3 activation after the DNA damaging agent cisplatin [35] as well as upregulation of a caspase-2 isoform that is anti-apoptotic [36]. The poor S-phase repair observed in melanoma in this study may indeed be a consequence of reduced or delayed apoptosis as a result of reduced XPC, but to confirm this further studies are required. Therefore, attenuation of GGR components XPC and DDB2 would also confer an anti-apoptotic phenotype in addition to the accumulation of DNA damage, both of which are key features of melanoma.

Transcriptome analysis of melanomas with low XPC revealed increased expression of transcripts involved in other DNA repair processes, in particular double strand break repair (DSBR). A similar study investigated transcript expression of approximately 500 cancer related genes in 472 FFPE primary melanomas and found overexpression of DNA repair genes, predominantly DSBR genes, was associated with patients that progressed on treatment and had shorter relapse-free survival [37]. An earlier analysis of whole genome transcript expression in 60 primary melanoma tumours found a DNA repair gene signature with high expression in tumours that progressed to metastatic disease [38]. Once again there was an over-representation of DSBR genes but none of the genes from the NER pathway were present in the gene signature. We postulate that the increased expression of other DNA repair pathways, particularly DSBR is a compensatory mechanism for reduced NER. DSBs can form when photoproducts are left unrepaired, providing more evidence for a link between high DSBR activity and low NER activity.

The high XPC expressing melanomas displayed significantly higher expression of immune response related transcripts. This supports a previous study that found a 46-gene expression signature containing immune response genes was predictive of better survival [39]. We endeavoured to confirm the relationship between low XPC expression and poor survival in independent published datasets. The most comprehensive was the TCGA data which showed a trend towards confirming the relationship between low XPC and poorer survival, but this requires confirmation in a large cohort of melanomas using a quantitative measure of XPC. All previous studies where XPC transcript expression levels were quantified by microarray analysis did not contain sufficient clinical information to determine survival from primary diagnosis or utilised considerable smaller cohorts.

The data reported herein indicates GGR deficiency plays a key role in melanoma and has the potential to produce informative biomarkers for melanoma stratification. We found that *XPC* deficiency is associated with an aggressive disease phenotype irrespective of disease stage. XPC is a DNA damage recognition protein, therefore deficiency is likely to play a key role in the accumulation of mutations in melanoma, and possibly development of treatment resistance and disease progression.

## MATERIALS AND METHODS

### Cell culture

Four melanoma cell lines were supplied by Prof Xu Dong Zhang: MM200, Sk-mel-28 Mel-RM and Me4405. The tumour status [40, 41] and p53 status [42] of each melanoma cell lines have been previously described. Human neonatal, medium (HEM1455 and HEMn-MP) and dark (HEMn-DP) pigmented epidermal melanocyte cell lines were purchased (Cascade Biologics, USA and ThermoFisher, USA). Cell line authentication was performed as previously described [16] and using GenePrint 10 (Promega, USA). Mycoplasma was tested and not detected at 6 month intervals using the MycoSEQ mycoplasma detection kit (Life Technologies, USA). Melanoma cell lines were cultured in 1x DMEM (Gibco, Life Technologies, USA) and melanocytes were cultured in Medium 254 (Gibco, USA) All cells were incubated at 37°C 5% CO_2_.

### UVB-irradiation

Cells were treated with 650J/m^2^ UVB in a BS-04 UV chamber (Dr. Gröbel UV-Elektronik GmbH, Germany). 650J/m^2^ was determined to be the UVB LD50 of HEMn-MP and was used for further analysis as it elicited DNA damage but not excessive apoptosis. Cell survival was quantified by trypan blue and/or flow cytometry using PE Annexin V Apoptosis Detection Kit 1 (BD Pharmingen). At 24hours after 650J/m^2^ the melanoma cell lines had variable cell survival compared to 50.9% in HEMn-MP and 60% in HEM1455 melanocyte cell lines. Cell survival at 24hours was 76% in MM200, 72.5% in Sk-mel-28 and 87.7% in Mel-RM. Me4405 had the lowest cell survival of 30.7% at 24 hours. There was no selection bias for alive intact cells for the remainder of the study. Alive, apoptotic and dead cells were included in all further analyses.

Quantification of UV-induced DNA damage: 6-4 PPs and CPDs were quantified by a flow cytometry protocol adapted from [28]. Primary antibodies used were 6-4 PPs (1:2000) or CPDs (1:1000) (Kamiya Biomedical). FITC-conjugated rabbit anti-mouse secondary antibody (Dako) and 10μg/ml propidium iodide (Sigma-Aldrich) were used for detection. Repair was analysed using a flow cytometer (BD FACSCanto II) by gating each of the cell cycle phases and quantifying the geometric mean fluorescence over time. The no UVB signal was subtracted for background normalisation. The fluorescent signal was then normalised to baseline (immediately after UVB) and results presented as percentage of repair.

### Cell cycle analysis

Cells were incubated in media containing BrdU (1:100, Life Technologies, USA) for 30 minutes; washed with PBS and resuspended in fresh media. At timepoints cells were fixed in ice cold 75% ethanol, washed with PBS + 50mM EDTA, resuspended and incubated in 0.5% Triton X-100 + 2M HCl at 22°C for 20 minutes. Cells were then washed with 0.1M Na2B4O7 (pH 9) followed by PBS and incubated with RNase A (Sigma-Aldrich) (100 μg/ml in PBS) at 37°C for 1 hour. Following this cells were washed with PBS-TB (1% bovine serum albumin + 0.25% Tween 20 in PBS) and resuspended in PBS-TB containing an Alexa-Fluor647-conjugated anti-BrdU antibody (1:200) (Life Technologies) for 1 hour. Cells washed with PBS-TB and resuspended in PBS containing 10μg/ml propidium iodide (Sigma-Aldrich). Cell cycle was analysed using a flow cytometer (BD FACSCanto II). To address the potential bias that high expressing XPC cells go into rapid apoptosis, Sub-G1 cell debris content was determined to be < 10% at 24hrs in all cell lines except MM200 (22.5%) and HeMn-DP (24.2%).

### Melanoma tumours

Formalin fixed paraffin embedded (FFPE) melanoma tumours; collected for diagnostic purposes at the Hunter Area Pathology Service, NSW, Australia between 2004 and 2009; were used for this study. The Hunter New England Area Health Service Human Ethics Committee approved the study. 196 tumours were identified with sufficient tissue (> 2mm width and length). All cases utilised had stage 2 or greater disease as either metastatic lymph nodes or primary melanomas greater than 2mm were used. RNA was successfully extracted from 157 of the 196 tumours (80%). Clinical information is summarised in Supplementary Table 1.

### Block biopsies and RNA extractions

Hematoxylin and eosin (H&E) stained slides for each block were examined to identify the area with the highest concentration of tumour tissue and minimal stromal and lymphocytic infiltration. A 2mm punch biopsy was taken through the block and RNA was extracted using Life Technologies RNA extraction kit as per manufacturer's instructions.

### Transcript and protein expression analysis

RNA was reverse-transcribed and relative expression (RE) was measured as described previously [16]. Relative expression (RE) was measured in triplicate and normalised to *GAPDH* and *β-actin* (ΔCt) using TaqMan gene expression assays (Applied Biosystems) and a ViiA7 system (Life Technologies). RE was calculated using 2^−ΔCt^

Protein fractions were obtained using the NucBuster protein extraction kit (Merck Millipore). Samples were loaded onto 4-20% TGX precast polyacrylamide gels (Bio-Rad Laboratories) and run at 150V (constant voltage) in Tris-Glycine buffer (25 mM Tris, 192mM glycine, 0.1% SDS). Proteins were transferred onto nitrocellulose and blocked in 5% skim milk for 1 hour at room temp. XPC and p53 proteins were detected using anti-XPC rabbit polyclonal antibody (H-300) (1:200; sc-30156 Santa Cruz Biotechnology, Inc.) and anti-p53 (Ab-2) (Pantropic) mouse monoclonal antibody (PAb1801) (1:1000; OP09 Calbiochem). anti-GAPDH EPR6256 (1:2500 ab128915 Abcam) was used as a loading control. Primary antibodies were incubated at 4°C overnight. Blots were washed in PBS-T then incubated for 1 hour at room temp with HRP-conjugated secondary antibodies (goat anti-rabbit 170-6515, goat anti-mouse 170-6516; Bio-Rad Laboratories). Blots were washed, then proteins detected using SuperSignal West Femto reagent (ThermoFisher Scientific) and imaged using the ChemiDoc MP system (Bio-Rad Laboratories). Image processing and densitometry analysis was perfomed using ImageJ for Mac OSX v1.49 (http://imagej.nih.gov/ij/). Data was normalised to GAPDH (p53) or total protein Ponceau staining and expressed as fold induction from baseline (two independent quantifications).

### Statistical analysis

Nonparametric Wilcoxon rank-sum tests were used to identify significantly different 6-4PP and CPD repair and transcript expression between melanoma and melanocyte cell lines, and to identify significant induction of transcripts.

Correlation between *XPC, DDB1,* and *DDB2* transcript expression and clinical parameters was performed using Spearman's Rho and Kendall's Tau tests. Confounding factors were further tested using multivariate linear regression. Kaplan-Meier survival analysis was performed by assigning each melanoma tumour to XPC+ (above median) or XPC- (below median) transcript expression. Breslow (generalised Wilcoxon) test was used to determine the Chi-squared and p-value for survival. Cox regression was used to correct for Breslow thickness and primary/metastatic status. The results of the second melanoma tumour dataset are in whole based upon data from 382 primary and metastatic melanoma tumours generated by the TCGA Research Network: http://cancergenome.nih.gov/. For the TCGA data the tumours in the lowest 10^th^ percentile and highest 10^th^ percentile of XPC mRNA expression were used for Kaplin-Meier survival analysis.

### Whole genome gene expression analysis

Whole genome gene expression analysis was performed using DASL assay and WGGEX V3 beadarrays (Illumina, San Diego, CA, USA). Data were cubic spline normalised using BeadStudio 2.0 software and analyses was performed using GeneSpring GX 11.0. The data have been deposited in NCBI Gene Expression Omnibus GSE59455 (http://www.ncbi.nlm.nih.gov/geo/query/acc.cgi?acc=GSE59455).

Data quality for the FFPE RNA was checked using principle component analysis (PCA) and outlying melanomas were removed before further analyses, resulting in 141 melanomas. Gene sets with significantly different transcript expression between XPC low (XPC-) and high XPC (XPC+) expression were determined using Mann-Whitney unpaired tests. Biological processes and gene sets over-represented were determined using the MSigDB [43] and Gene Ontology (GO) terms [44]. GO terms with *p*-values < 1 × 10^−4^ and false discovery rates (FDR) q-values < 0.001 were used.

## SUPPLEMENTARY MATERIALS FIGURE AND TABLE


