# Supplementary material for: Repair of UVB-induced DNA damage is reduced in melanoma due to low XPC and global genome repair
Source: Oncotarget. 2016 Jul 28;7(38):60940–53. doi: 10.18632/oncotarget.10902 (PMC5308628; doi:10.18632/oncotarget.10902)
Supplement: Supplementary file 1 [file oncotarget-07-60940-s001.pdf]

Repair of UVB-induced DNA damage is reduced in melanoma due to low XPC and global genome repair

Supplementary Material

Supplementary Table 1. Clinical parameters

Clinical Parameter	Total Patient No. (%)
**Total**	157 (100)
**Sex**	
Female	48 (30.6)
Male	109 (69.4)
**Age at 1st Diagnosis**	
Mean (range)	65.8 (23.3 - 94.5)
Unknown	15 (9.6)
**Survival (weeks)**	
Mean (range)	206.6 (3.1 - 1418)
Alive	17 (10.8)
**Breslow Thickness**	
Mean (range)	5.3 (0.4 - 33)
No. Unknown	62 (39.5)
**Solar Elastosis**	
None	8 (5.1)
Mild	25 (15.9)
Moderate	17 (10.8)
Severe	42 (26.8)
Unknown	65 (41.4)
**Clark's Level (Stage)**	
1	16 (10.2)
2	47 (29.9)
3	47 (29.9)
4	21 (13.4)
No. Unknown	26 (16.6)
**Weeks Local to Distal Metastasis**	
Mean (range)	121 (5.6 - 343.4)
No. Unknown	66 (42)

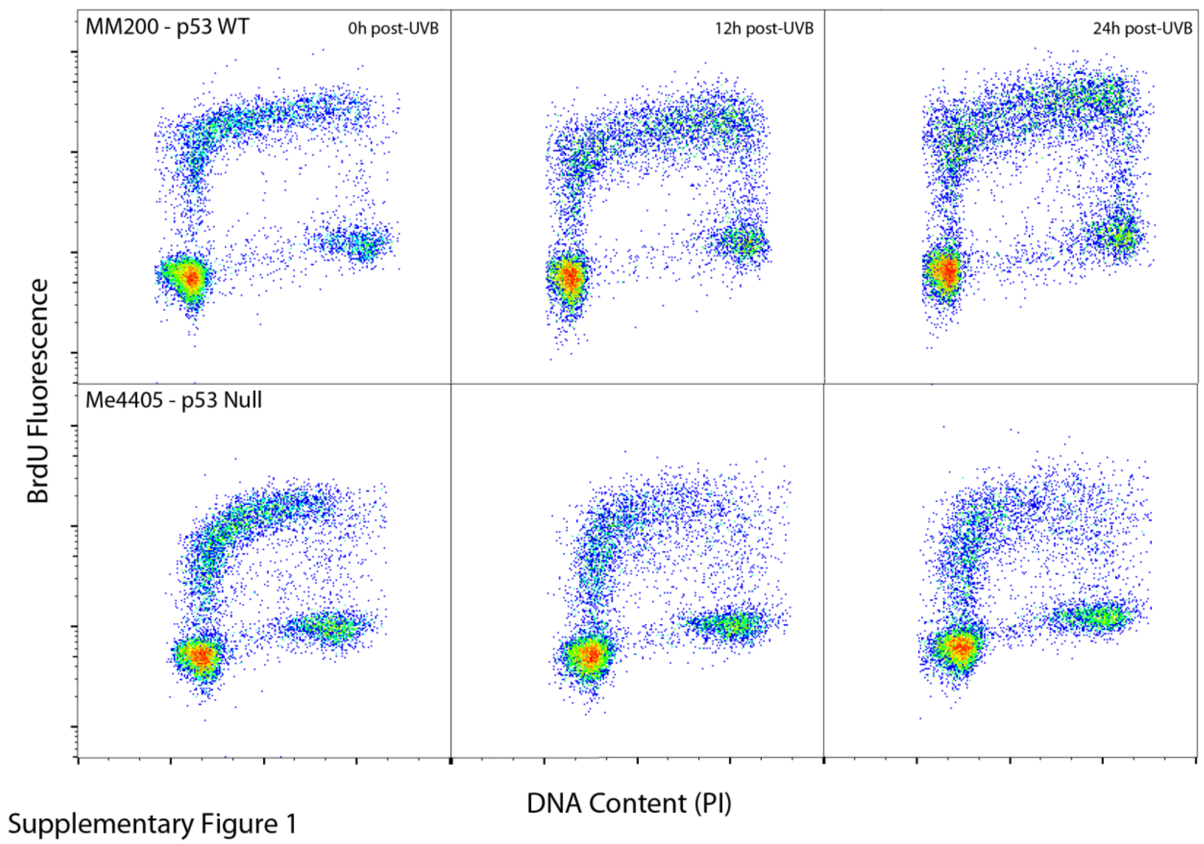

Supplementary Figure S1. Cell cycle of melanoma cell lines after 650J/m^2 UVB: The cell cycle of p53 WT melanoma cell line MM200 and p53 null cell line Me4405 were analysed after 650J/m^2 UVB using BrdU and PI. Cells were treated with BrdU before UVB treatment and cells in each phase of the cell cycle were examined prior to and then 12 and 24 hours post UVB.
